# Dispersive Micro-Solid Phase Extraction Using a Graphene Oxide Nanosheet with Neocuproine and Batocuproine for the Preconcentration of Traces of Metal Ions in Food Samples

**DOI:** 10.3390/molecules28104140

**Published:** 2023-05-17

**Authors:** Barbara Feist

**Affiliations:** Institute of Chemistry, University of Silesia, Szkolna 9, 40-006 Katowice, Poland; barbara.feist@us.edu.pl

**Keywords:** graphene oxide, metal ions, neocuproine, batocuproine, dispersive micro-solid phase extraction, preconcentration, ICP-OES

## Abstract

A dispersive micro-solid phase extraction (Dµ-SPE) method for the preconcentration of trace metal ions (Pb, Cd, Cr, Mn, Fe, Co, Ni, Cu, Zn) on graphene oxide with the complexing reagents neocuproine or batocuproine is presented here. Metal ions form cationic complexes with neocuproine and batocuproine. These compounds are adsorbed on the GO surface via electrostatic interactions. The factors affecting the separation and preconcentration of analytes such as pH, eluent (concentration, type, volume), amount of neocuproine, batocuproine and GO, mixing time, and sample volume were optimized. The optimal sorption pH was 8. The adsorbed ions were effectively eluted with 5 mL 0.5 mol L^−1^ HNO_3_ solution and determined by the ICP-OES technique. The preconcentration factor for the GO/neocuproine and GO/batocuproine in the range 10–100 and 40–200 was obtained for the analytes, with detection limits of 0.035–0.84 ng mL^−1^ and 0.047–0.54 ng mL^−1^, respectively. The method was validated by the analysis of the three certified reference materials: M-3 HerTis, M-4 CormTis, and M-5 CodTis. The procedure was applied to determine metal levels in food samples.

## 1. Introduction

Modern industrial development has brought significant benefits to people; on the other hand, it also produces large amounts of waste. Most of this waste is made up of harmful substances—especially heavy metals; these are the cause of water, soil, and atmospheric pollution. Environmental pollution with heavy metals is a severe problem due to their toxicity to humans, animals, and the natural environment [1]. Heavy metal ions do not decompose in the environment, but they can accumulate in living organisms, causing many diseases, e.g., of the brain, lungs, kidneys, liver, and reproductive organs. Ultimately, they can cause death [2]. Lead, cadmium, nickel, and mercury are the most dangerous heavy metals. Copper and zinc in trace amounts are necessary because they are important in biological processes; however, their higher concentrations can be harmful and hazardous to health [3]. Therefore, it is important to monitor the content of heavy metals in environmental samples and foodstuffs.

Modern analytical chemistry offers various spectroscopic techniques enabling the determination of analytes at trace or ultra-trace levels. Nevertheless, the direct determination of such small amounts of analytes with the use of spectroscopic techniques, i.e., atomic absorption spectrometry (FAAS, ETAAS), inductively coupled plasma optical emission spectrometry (ICP-OES) or inductively coupled plasma mass spectrometry (ICP-MS) can be problematic. Although the abovementioned techniques can be considered sensitive, reproducible, and precise, the complexity of biological and environmental matrices and low concentrations of analytes lead to the application of additional isolation and/or preconcentration procedures [4].

Considering contemporary trends in modern chemistry, i.e., the reduction of toxic organic reagents and solvents, decreases in costs and the time of analysis, environmental friendliness, and the simplicity of analytical procedures, solid phase extraction (SPE) seems to be the most applicable method [5]. Moreover, the main aim of solid phase extraction is analyte isolation and preconcentration; this technique achieves high preconcentration factor values, reflecting decreases in detection limit values. In addition to classic SPE, its variants are also used—dispersive solid phase extraction (DSPE) [6] and dispersive micro-solid phase extraction (Dµ-SPE) [7].

To ensure the quantitative determination of analytes, the proper choice of adsorbent material plays an important role in SPE. In recent years, carbon sorbents have been very popular and used often, such as graphene [8,9,10], graphene oxide (GO) [11,12,13], reduced graphene oxide (rGO) [14,15,16], magnetic graphene oxide (MGO) [17,18,19], carbon nanotubes (CNT) [20,21,22], fullerenes [23,24,25], carbon nanohorns (CNHs) [26], carbon nanofibers (CNFs) [27,28], and activated carbon (AC) [29,30,31].

**Figure 1:**
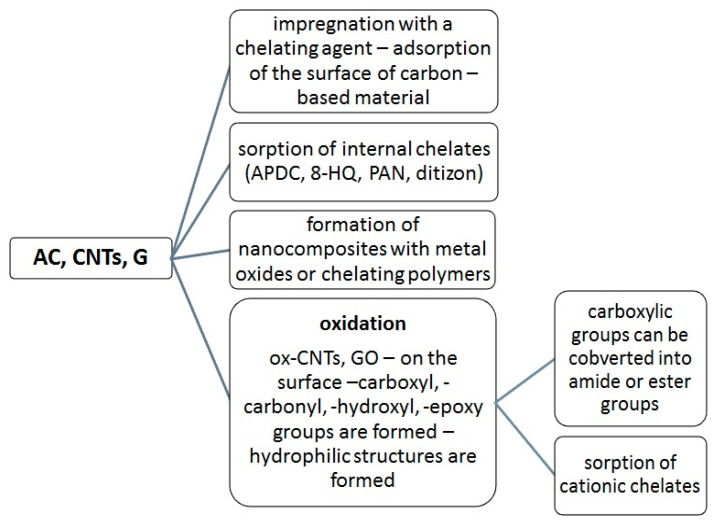
Modification of carbon-based materials’ surface.

The disadvantage of most of these carbon materials is their hydrophobic character, which makes the adsorption of metal species impossible. In order to make the adsorption of metal ions possible, the formation of hydrophobic complexes—usually inner chelate complexes with analytes—is required. In that case, hydrophobic complexes are adsorbed on the sorbent surface by van der Waals forces or hydrophobic interactions. The adsorbents can be modified, i.e., by impregnation with a chelating agent. This modification can be performed by passing the chelating agent through a column packed with adsorbents [12] or, more frequently, by mixing the adsorbent with a chelating agent for a specific period of time, i.e., AC with rhodamine 6G [32] or PAN [33], or CNTs with PAN [22,34]. It should be underscored here that carbon-based adsorbents with surfaces subjected to a modification that changes their character from hydrophobic to hydrophilic are used more frequently in analytical chemistry—this is the case for graphene oxide. Oxidized adsorbents can be also modified by the formation of amide or ester bonds; this type of chemical modification is called functionalization [35,36,37,38,39,40]. Moreover, composites of carbonaceous materials with metal oxides, e.g., MnO_2_ [41] or magnetic composites [42], are applied in SPE procedures. The methods used for the modification of carbon-based adsorbents are presented in Figure 1.

A less-frequently used method of modifying the oxidized surface of carbon materials, e.g., GO, is the sorption of cationic chelates. Cationic complexing compounds include 1,10-phenanthroline and its derivatives. These contain nitrogen atoms possessing a lone pair of electrons, thanks to which they can effectively bind metal ions, forming a cationic metal complex. These cationic complexes can be adsorbed onto the negatively charged surface of the adsorbent. Metal ions complexed with 1,10-phenanthroline derivatives are adsorbed onto the GO via electrostatic interactions and van der Waals forces—particularly π-electron interactions. Earlier studies developed a method based on batophenanthroline (4,7-diphenyl-1,10-phenanthroline) to selectively improve the determination of heavy metal ions [43]. This work used two other 1,10-phenanthroline derivative compounds containing methyl groups: 2,9-dimethyl-1,10-phenanthroline (neocuproine) and 2,9-dimethyl-4,7-diphenyl-1,10-phenanthroline (batocuproine) as well as the carbon adsorbent graphene oxide. The study aimed to investigate the effect of the 1,10-phenanthroline derivatives on the preconcentration of trace amounts of heavy metal ions on GO.

## 2. Results and Discussion

Dispersive micro-solid phase extraction (Dµ-SPE) was used in the analytical procedure presented. To determine the optimal conditions for the sorption of the cationic complexes of neocuproine and batocuproine with metal ions on graphene oxide, the following parameters were tested: pH, type and concentration of eluent, amount of sorbent, complexing agent mixing time, and sample volume. Finally, the influence of the inorganic matrix on the sorption performance was also investigated. All experiments were carried out in four replicates.

### 2.1. Influence of the Analytical Conditions on the Sorption Process

#### 2.1.1. Effect of pH

**Figure 2:**
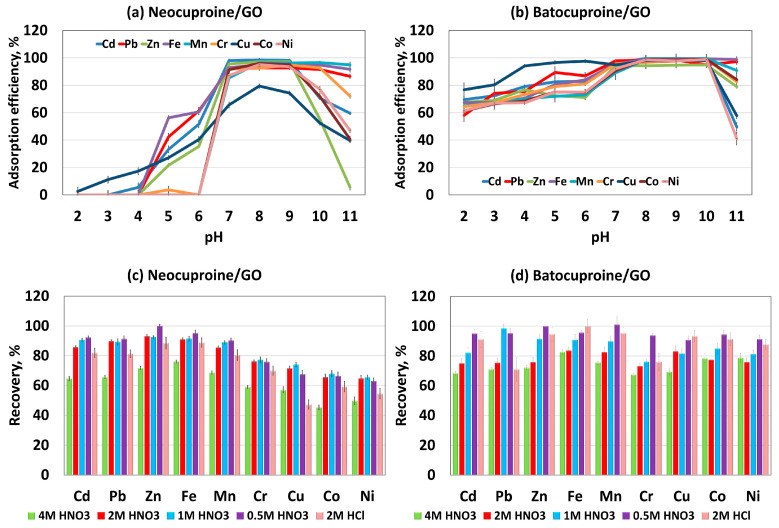
Adsorption of metal ions on GO sorbent in Dµ-SPE: (**a**,**b**) the influence of pH (**c**,**d**) the effect of the concentration and type of eluent on the recovery of metal ions.

The pH of the solution is a critical parameter as it significantly influences the adsorption of metal ions. The effect of pH on the sorption of cationic metal complexes with (i) neocuproine and (ii) batocuproine was tested in 2–11. The results are shown in Figure 2a for the system with neocuproine and Figure 2b for the system with batocuproine. When neocuproine was used, the sorption of cationic metal complexes was almost absent in the pH range 1–4. Only at pH 4 was an increase in the sorption of metal ion complexes observed—except for Cr and Ni. In the pH range of 7–9, they absorbed almost all metal ions with more than 90% recovery. The exception was the Cu complex, whose sorption reached a maximum value of no more than 80%. Above pH 9, a decrease in the sorption of metal complexes was observed, except for the Mn, Fe, and Pb complexes. When batocuproine was used as a chelating agent, the sorption of all ions above pH two was observed, with at least 60% recovery. In the pH range of 7–10, all cationic complexes of batocuproine with metal ions sorbed on GO with more than 90% recovery. At a pH above 10, a slight decrease in sorption was observed. Therefore, a pH value of 8 was used as the optimum pH for further investigations of neocuproine and batocuproine.

#### 2.1.2. Study of Desorption Conditions

The type and concentration of the acids required for the elution of the metal chelates adsorbed on the GO were investigated to optimise the elution conditions. The effect of nitric acid and hydrochloric acid was tested with the concentrations in Figure 2c,d. In the elution of metal ion complexes with neocuproine, the desorption efficiency for Cr, Cu, Co, and Ni was 40–80%, regardless of the eluent used. Nitric acid at a concentration of 0.5 mol L^−1^ was the most effective for eluting the remaining analytes. Further studies on the sorption of metal ions using neocuproine focused on determining Pb, Cd, Zn, Fe, and Mn. Using complexes with batocuproine, all adsorbed ions could be eluted from the GO with more than 90% recovery using 0.5 mol L^−1^ nitric acid. The least effective eluent in each case was nitric acid at a concentration of 4 mol L^−1^. The effect of the 0.5 mol L^−1^ nitric acid volume on the desorption efficiency was also investigated; 5 mL or 10 mL of the eluent was used for the tests. No difference in elution efficiency was found. Therefore, 5 mL of nitric acid was used in further studies. This amount was sufficient to determine the analytes in the samples using the ICP-OES technique.

#### 2.1.3. Effect of the Amount of Adsorbent and Chelating Agent

To optimize the amount of sorbent, the effect of the amount of GO was tested in the range of 1–4 mg. Using 1 mg GO, slightly poorer recoveries of the analytes were obtained in each case, i.e., below 90. In the range of 2–4 mg GO, the recoveries were above 90% and comparable. Therefore, 2 mg of GO was used for further tests.

In order to optimize the amount of the chelating agent, the effect of different volumes of the solution of (i) neocuproine with a concentration of 0.01 mol L^−1^ (in ethanol) in the range of 0.5–2 mL and (ii) batocuproine with a concentration of 0.1% (in ethanol), in the range of 0.2–2 mL, was studied. The amount of neocuproine had no significant effect on the recovery of metal ions. Using only 0.5 mL of this compound reduced the recovery of Mn ions to 80%. It was assumed that 1 mL was the optimal amount. When batocuproine was used as a chelating agent, more than 90% of all ions were recovered with 0.2 mL. Therefore, this amount was considered sufficient for further studies.

The concentration experiments were also carried out without a chelating agent. In this case, the results were significantly less reproducible, and the recoveries were somewhat lower.

#### 2.1.4. Effect of the Time of Contact and Sample Volume

**Figure 3:**
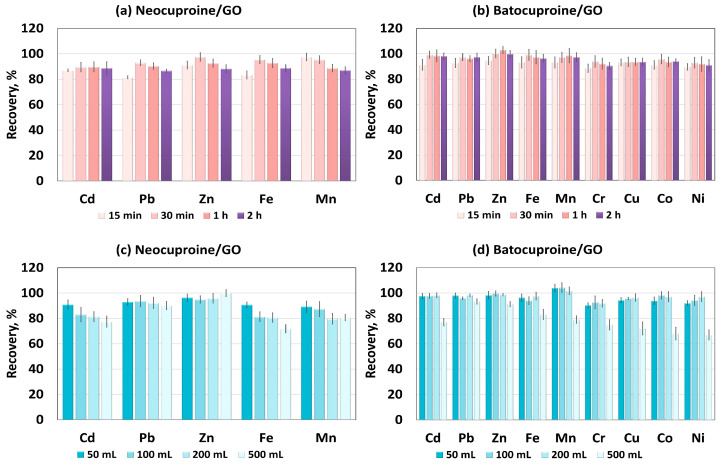
Adsorption of metal ions on GO sorbent in Dµ-SPE: (**a**,**b**) influence of sorption time, (**c**,**d**) influence of volume sample.

Two critical parameters affecting sorption are the mixing time and sample volume. Too short a time can be the cause of ineffective sorption. On the other hand, too long a mixing time not only prolongs the duration of the analytical procedure but can also lead to the desorption of metal ions, reducing recovery. The influence of mixing time on the sorption efficiency of metal chelates on GO was investigated in a range of 15–120 min. The results for neocuproine and batocuproine are shown in Figure 3a,b, respectively. It can be concluded that when using both neocuproine and batocuproine, mixing the samples for 30 min was sufficient to achieve recoveries above 90%.

The influence of sample volume on the adsorption efficiency of the cationic metal chelates on GO was tested in the 50–500 mL range. Increasing the sample volume will result in higher preconcentration factors (PF) and, thus, lower detection limits (DL). The results obtained are shown in Figure 3c,d. The sample volume had no significant effect on the recovery of Pb and Zn in the case of both neocuproine and batocuproine. When batocuproine was used as a chelating agent for all the other analytes, the recovery was above 90% at a volume of 200 mL. Worse results were obtained with neocuproine as a chelating agent. In the case of Cd, Mn, and Fe, the recovery of the analytes decreased significantly with increases in sample volume. The obtained preconcentration factors for the analytes are listed in Table 1.

#### 2.1.5. Interferences Study

The influence of coexisting and potential interfering ions on the sorption efficiency of cationic complexes of analytes with neocuproine and batocuproine on GO was also investigated. Coexisting ions are present in addition to the analytes in the actual samples and can interfere with the separation and determination of the analytes. The influence of these ions was tested for each of the analytes individually. The interferent to analyte ratio and the results are shown in Table 2. The results show that the coexisting ions did not significantly influence the determined metal ions after their sorption on GO. Analyte recoveries were greater than 90% in each case.

### 2.2. Analytical Performance

To evaluate the method’s applicability to the analysis of metal ions, the linearity range of the methods for each analyte and the detection limits, quantification limits, precision, and accuracy were determined. The detection limits of the analytes for the ICP-OES technique were determined as described in the article [44]. Lower detection limits were obtained for the batocuproine system. The results are shown in Table 2. The precision of the method was determined in eight replicates for three different concentrations of analytes (5 ng mL^−1^, 10 ng mL^−1^, and 20 ng mL^−1^). The relative standard deviation did not exceed 4%. Three certified reference materials were analyzed to verify the accuracy of the method. The results obtained are shown in Table 3. Based on these, it can be concluded that there was no significant difference between the certified and received values.

A comparison of the described method with recently published methods for the determination of metal ions after their concentration using Dµ-SPE is presented in Table 4. In the methods developed using cationic complexes of neocuproine and batocuproine, significantly lower detection limits and higher preconcentration factors for selected metal ions were obtained than those found with other presented methods. The influence of methyl and phenyl substituents in the aromatic system for 1,10-phenanthroline derivatives is noticeable. The results obtained from the conducted studies on the effectiveness of the cationic adsorption of complexes of three 1,10-phenanthroline derivatives, i.e., (i) batophenanthroline (4,7-diphenyl-o-phenanthroline), (ii) neocuproine (2,9-dimethyl-o-phenanthroline), and (iii) batocuproine (2,9-dimethyl-4,7-diphenyl-o-phenanthroline) with nine metal ions (Cd, Co(II), Cu(II), Ni(II), Pb(II), Zn, Cr(III), Mn(II), and Fe(III)) showed that the sorption efficiency of cationic metal complexes decreases in the following order: batocuproine > batophenanthroline > neocuproine. All three complex compounds that form cationic chelates with metal ions—i.e., the neocuproine and batocuproine presented in this paper and the batophenanthroline presented in [43]—can be used in the multi-element analysis of real samples. A large dynamic linear range characterized all the proposed methods. However, the most effective method was the one using batocuproine. We then obtained the lowest detection limits. Using batocuproine and neocuproine, we were able to concentrate Pb and Zn ions in large sample volumes (500 mL) with a recovery of over 90%. In turn, the maximum sample volume for the system with bathophenanthroline was 200 mL for each analyte.

### 2.3. Application

The methods were successfully used to determine metal ions in pork liver and kidney. The accuracy of the process was checked by the standard addition method. The analytical results are shown in Table 5. The analytes’ recoveries were in the 94–102% range. The relative standard deviation did not exceed 3%. The results show that the method was accurate. The results for neocuproine and batocuproine as metal ion chelating agents were comparable.

## 3. Materials and Methods

### 3.1. Instruments

An inductively coupled plasma optical emission spectrometer, ICP-OES model Spectroblue Spectro Analytical Instruments GmbH (Kleve, Germany) was used to determine the concentrations of all the metal ions. The exact operating parameters of the spectrometer are given in the article [44]. The selected wavelengths for the individual metal ions were Cr—267.716 nm, Mn—257.611 nm, Fe—259.941 nm, Co—238.892 nm, Ni—231.604 nm, Cu—219.958 nm, Zn—213.856 nm, Cd—228.802 nm, and Pb—220.353 nm.

An Ethos Up microwave digestion system Milestone (Sorisole, Italy) equipped with a Teflon high-pressure reaction vessel was used to digest the samples and the certified reference materials.

### 3.2. Reagents and Solutions

Metal stock solutions (Cr(III), Mn(II), Fe(III), Co(II), Ni(II), Cu(II), Zn(II), Cd(II), and Pb(II); 1000 mg L^−1^ in 1% HNO_3_) neocuproine and batocuproine were purchased from Merck (Darmstadt, Germany). Graphite powder was obtained from Sigma-Aldrich (St. Louis, MI, USA). Sulfuric acid (98%, p.a.), nitric acid (65%, p.a.), hydrochloric acid (35–38%, p.a.), hydrogen peroxide (30%, p.a.), ammonium hydroxide solution (25%, p.a.), ethanol (96%, p.a.), boric acid (p.a.), disodium tetraborate (p.a.), sodium nitrate (p.a.), potassium permanganate (p.a.), and all of the salts (p.a.) used in the study of potentially coexisting ions influence were bought from Avantor (Gliwice, Poland). The certified reference materials MODAS-3 Herring Tisue (M-3 HerTis), MODAS-4 Cormorant Tissue (M-4 CormTis), and MODAS-5 Cod Tisue (M-5 CodTis) were purchased from Consortium MODAS LGC Standards (Warsaw, Poland). The experiments were carried out using ultrapure water provided with a Milli-Q system (Millipore, Molsheim, France).

A pH 8 borate buffer solution was prepared by mixing an appropriate amount of boric acid with an appropriate amount of disodium tetraborate. Neocuproine (0.01 mol dm^−3^) and batocuproine (0.1%) were prepared in ethanol (96%).

### 3.3. Synthesis and Characterization of GO

GO was obtained by the Hummers’ method using concentrated sulfuric acid, potassium permanganate, and sodium nitrate [51]. The GO was characterized by X-ray photoelectron spectroscopy (XPS), powder X-ray diffraction (XRD), and Raman spectroscopy [52].

As presented in the article [52], XPS shows the presence of oxygen groups on the surface of the GO compared to the graphite from which the GO was obtained. The C 1s spectrum of graphite shows the main peak at 284.4 eV due to the graphitic carbon, whereas the spectrum of the GO also revealed four other peaks at 285.8, 286.9, 288.1, and 289.5 eV, assigned to C−OH, C−O−C, C=O, and O−C=O. The C 1s spectrum of the GO also showed a main peak at 284.6 eV, assigned to the non-oxidized carbon C-C/C-H. The O 1s spectrum of the GO showed three peaks at 531.2, 532.4, and 533.6 eV, assigned to O−C=O (carboxyl and carbonyl groups), C-OH/C-O-C (hydroxyl and epoxy groups), and H_2_O/C-OH (carboxyl group).

### 3.4. Sample Preparation

Certified reference materials. 200 mg of Herring Tisue, Cormorant Tissue, and Cod Tisue were digested in 5 mL of concentrated nitric acid using microwave-assisted digestion. Mineralization was carried out for 30 min in Teflon (PTFE) vessels with a capacity of 100 mL at a temperature of 210 °C, a pressure of 50 bar, and a power of 1800 W. After cooling, the obtained solution was diluted to a volume of about 50 mL. Next, the samples were prepared using the preconcentration procedure Dµ-SPE/ICP-OES. The same procedure was used for the blank solutions.

Pork liver and pork kidney. The pork liver and pork kidney were purchased from a supermarket in Poland. The samples were lyophilized, ground in a high-speed rotor mill, and sieved through sieves with a mesh size of 500 µm. Next, the procedure was similar to the certified reference materials.

### 3.5. Dµ-SPE Procedure

In the first step, the GO was dispersed in high-purity water to obtain a homogenous suspension with a concentration of 2 mg mL^−1^. Next, 1 mL of the GO suspension was transferred to 50 mL of the analyzed solution containing (i) 1 mL of neocuproine solution or (ii) 0.2 mL of batocuproine solution and metal ions. Afterwards, the solution’s pH was adjusted to 8 with borate buffer. The sample solutions were stirred on a magnetic stirrer for 30 min. After this time, the solutions were passed through cellulose filters under reduced pressure. The adsorbed metal ions were eluted with 5 mL of 0.5 mol L^−1^ HNO_3_. The analytes were determined using the ICP-OES technique.

## 4. Conclusions

Based on the conducted research, it was found that the use of neocuproine and batocuproine as chelating agents for the initial preconcentration of trace amounts of Pb(II), Zn(II), Cd(II), Fe(III), Mn(II), Cr(III), Cu(II), Co(II), and Ni(II) in food samples before their determination with the ICP-OES technique is effective and gives replicable results. Thus, this broadens the range of possible GO applications for analysing food samples. An undeniable advantage is the lack of influence of the inorganic matrix on the analytes being determined. Cationic complexes of these metals adsorbed on graphene oxide can be easily eluted with 0.5 mol L^−1^ of nitric acid. These methods are fast, simple, economical, and environmentally friendly.

## Figures and Tables

**Table 1:** Analytical performance characteristics of the Dµ-SPE/ICP-OES method for metal ion determination.

Analyte/ R^2^	PF	Linear Range, ng mL^−1^	DL (3σ), ng mL^−1^	QL (3σ), ng mL^−1^	Determined, ng mL^−1^, *n* = 8 RSD, %		
					5 ng mL^−1^	10 ng mL^−1^	20 ng mL^−1^
	Neocuproine/GO						
Cd 0.9995	10	1–1000	0.24	0.79	4.56 ± 0.12 2.71	9.37 ± 0.24 2.71	18.49 ± 0.31 1.67
Pb 0.9998	100	1–1000	0.22	0.73	4.76 ± 0.15 3.15	9.27 ± 0.22 2.43	18.40 ± 0.72 3.89
Zn 0.9997	100	0.5–1000	0.035	0.12	4.74 ± 0.10 2.19	9.18 ± 0.33 3.59	18.86 ± 0.69 3.65
Fe 0.9989	10	3–1000	0.84	2.80	4.89 ± 0.17 3.48	9.38 ± 0.35 3.73	18.34 ± 0.57 3.11
Mn 0.9984	20	1–1000	0.17	0.57	4.83 ± 0.14 2.90	9.42 ± 0.35 3.71	18.21 ± 0.39 2.14
	Batocuproine/GO						
Cd 0.9995	40	0.5–1000	0.047	0.16	4.83 ± 0.22 1.72	9.68 ± 0.19 1.83	18.83 ± 0.09 1.75
Pb 0.9998	100	0.5–1000	0.089	0.30	4.84 ± 0.36 1.72	9.80 ± 0.17 1.69	19.13 ± 0.07 1.28
Zn 0.9997	100	0.5–1000	0.083	0.27	4.78 ± 0.49 2.34	9.48 ± 0.34 3.21	18.65 ± 0.08 1.43
Fe 0.9989	40	0.5–1000	0.12	0.40	4.76 ± 0.29 1.38	9.45 ± 0.25 2.37	93.06 ± 0.02 1.63
Mn 0.9984	40	0.5–1000	0.13	0.43	4.69 ± 0.42 1.98	9.48 ± 0.29 2.78	18.71 ± 0.12 2.33
Cr 0.9987	40	0.5–1000	0.077	0.25	4.70 ± 0.45 2.10	9.62 ± 0.23 2.20	18.98 ± 0.14 2.60
Cu 0.9985	40	2–1000	0.54	1.78	4.65 ± 0.52 2.41	9.28 ± 0.30 2.79	18.32 ± 0.12 2.11
Co 0.9995	40	0.5–1000	0.071	0.23	4.61 ± 0.51 2.36	9.29 ± 0.29 2.67	18.51 ± 0.17 3.20
Ni 0.9991	40	1–1000	0.15	0.50	4.60 ± 0.43 1.97	9.19 ± 0.25 2.26	18.39 ± 0.08 1.53

**Table 2:** Influence of coexisting ions on the recovery analytes. The concentration of analytes was 10 ng mL^−1^, *n* = 4.

Interferent Ions	Al	Ba	Sr	Mg	Ca	K	Na
Interferent to analyte ratio	1:500	1:1000	1:1000	1:10,000	1:20,000	1:20,000	1:20,000
Recovery,%	Neocuproine/Go						
Cd	92.5 ± 2.6	98.1 ± 3.7	90.2 ± 1.8	95.6 ± 4.0	96.9 ± 2.3	92.2 ± 1.9	90.2 ± 2.1
Pb	93.7 ± 2.7	95.6 ± 3.3	96.7 ± 4.2	91.1 ± 3.5	91.4 ± 2.1	90.9 ± 2.3	99.0 ± 1.5
Zn	100 ± 3	92.1 ± 1.5	100 ± 3	92.8 ± 3.9	97.1 ± 2.6	99.1 ± 1.7	100 ± 3
Fe	99.7 ± 1.4	92.6 ± 1.7	92.6 ± 3.7	97.3 ± 2.3	99.2 ± 2.8	97.3 ± 3.2	98.4 ± 3.3
Mn	100 ± 2	93.6 ± 1.8	94.9 ± 3.4	92.5 ± 2.7	92.4 ± 1.3	100 ± 3	98.2 ± 1.8
	Batocuproine/GO						
Cd	94.6 ± 1.1	94.1 ± 2.4	90.8 ± 2.9	92.5 ± 1.9	93.2 ± 2.2	93.2 ± 3.1	91.5 ± 3.5
Pb	98.5 ± 2.4	91.4 ± 2.8	92.0 ± 2.8	95.2 ± 1.9	101 ± 3	96.2 ± 2.9	94.7 ± 3.5
Zn	97.0 ± 1.7	93.8 ± 2.9	92.9 ± 2.1	99.7 ± 2.3	102 ± 3	103 ± 3	93.8 ± 3.2
Fe	93.2 ± 2.8	93.3 ± 2.6	103 ± 3	95.8 ± 2.6	101 ± 3	97.5 ± 1.6	98.1 ± 2.4
Mn	91.9 ± 0.7	92.0 ± 2.5	90.8 ± 2.8	98.9 ± 0.8	99.1 ± 0.9	99.5 ± 1.5	96.5 ± 3.1
Cr	96.5 ± 2.9	94.5 ± 2.8	98.9 ± 2.4	91.5 ± 2.5	97.0 ± 2.4	90.8 ± 3.2	96.8 ± 2.3
Cu	92.6 ± 2.5	93.7 ± 2.7	91.3 ± 3.1	92.7 ± 0.7	98.9 ± 2.5	94.2 ± 1.9	92.8 ± 2.1
Co	93.5 ± 1.4	96.7 ± 2.5	93.7 ± 2.2	96.4 ± 1.7	94.4 ± 1.1	94.4 ± 2.6	91.6 ± 2.9
Ni	95.1 ± 1.1	69.5 ± 2.7	92.9 ± 2.4	91.3 ± 2.3	95.0 ± 1.6	93.9 ± 2.8	91.4 ± 3.2

**Table 3:** Analysis of the certified reference materials M-3 HerTis, M-4 CormTis, and M-5 CodTis by ICP-OES, *n* = 4.

Analyte	Certified Value, mg kg^−1^ (ng g^−1^) *	Found, mg kg^−1^ (ng g^−1^) *	Recovery, %	RSD, %	Found, mg kg^−1^ (ng g^−1^) *	Recovery, %	RSD, %
	M-3 HerTis	Neocuproine/GO			Batocuproine/GO		
Fe	119 ± 13	118 ± 4	99.2	3.4	117 ± 6	98.3	5.1
Mn	5.78 ± 0.61	5.48 ± 0.23	94.8	4.2	5.63 ± 0.31	97.4	5.5
Zn	111 ± 6	110 ± 5	99.0	4.5	107 ± 3	96.4	2.8
Cd	325 ± 30 *	319 ± 17 *	98.2	5.3	311 ± 9 *	95.7	2.9
Pb	104 ± 13 *	99 ± 6 *	95.2	6.0	102 ± 5 *	98.1	4.9
Cu	3.19 ± 0.22	-	-	-	3.14 ± 0.08	98.4	2.5
Co	81 ± 12 *	-	-	-	77 ± 4 *	95.1	5.2
Cr	900 ± 110 *	-	-	-	870 ± 33 *	96.7	3.8
Ni	316 ± 49 *	-	-	-	298 ± 11 *	94.3	3.7
	M-4 CormTis	Neocuproine/Go			Batocuproine/GO		
Fe	280 ± 16	263 ± 12	93.9	4.6	270 ± 14	96.4	5.2
Mn	2.16 ± 0.17	2.07 ± 0.09	95.8	4.3	2.09 ± 0.12	96.8	5.7
Zn	63.3 ± 3.5	58.4 ± 2.6	92.3	4.4	61.8 ± 2.9	97.6	4.7
Pb	2.33 ± 0.28	2.19 ± 0.08	94.0	3.6	2.27 ± 0.12	97.4	5.3
Cd	17.2 ± 2.1 *	16.6 ± 0.80 *	96.5	4.8	17.1 ± 1.1 *	99.4	6.4
Cu	19.5 ± 1.2	-	-	-	18.7 ± 0.6	95.9	3.2
Co	41.0 ± 0.28 *	-	-	-	40.7 ± 0.23 *	99.3	0.6
	M-5 CodTis	Neocuproine/GO			Batocuproine/GO		
Fe	13.2 ± 1.1	12.4 ± 0.18	94.0	1.5	12.6 ± 0.23	95.5	1.8
Mn	921 ± 75	873 ± 37	94.8	4.2	881 ± 32	95.7	3.6
Zn	20.1 ± 1.1	19.7 ± 0.80	98.0	4.1	19.3 ± 0.7	96.0	3.6
Cd	5 *	4.9 ± 0.32 *	98.0	6.5	4.7 ± 0.27 *	94.0	5.7
Pb	45 *	44 ± 1 *	97.8	2.3	43 ± 2 *	95.6	4.7
Cu	1.38 ± 0.09	-	-	-	1.34 ± 0.04	97.1	3.0
Co	14 *	-	-	-	12.9 ± 0.43 *	92.1	3.3
Cr	201 *	-	-	-	189 ± 4 *	94.0	2.1
Ni	136 *	-	-	-	124 ± 3 *	91.2	2.4

* Found in ng g^−1^.

**Table 4:** Analytical methods for the determination of metal ions using carbon nanomaterials in Dµ-SPE.

Adsorbent	Analytes	Technique	Eluent Volume, mL	Volume max, mL	PF	DL, ng mL^−1^	Ref.
CNT-BMBATT ^1^	Cd(II), Pb(II)	FAAS	2	100	200	0.08, 0.1	[45]
CNT-IL ^2^	Pb(II), Ni(II)	ETAAS	0.4	10	25	0.05, 0.1	[46]
GO-Fe_3_O_4_@PTh ^3^	Zn(II)	FAAS	2	30	60	0.08	[47]
GO-SiO_2_	Cd(II), Pb(II), Zn(II), Cr(III), Cu(II), Co(II), Ni(II)	FAAS	1	10	10	5.8–23	[48]
GO-Fe_3_O_4_@NpSH ^4^	Cd(II)	ETAAS	0.2	1	5	0.01	[49]
GO-Fe_3_O_4_@BMIM ^5^	Cd(II), Pb(II), Zn(II), Cr(III), Cu(II), Co(II)	ICP-OES	0.5	25	50	0.1–1	[50]
GO/Batophenanthroline	Cd(II), Pb(II), Zn(II), Cr(III), Fe(III), Mn(II)	ICP-OES	4	200	50	0.06–0.25	[43]
GO/Neocuproine	Pb(II), Zn(II), Mn(II) Cd(II), Fe(III)	ICP-OES	5	500 100 50	100 20 10	0.035–0.84	This work
GO/Batocuproine	Pb(II), Zn(II), Cd(II), Fe(III), Mn(II), Cr(III), Cu(II), Co(II), Ni(II)	ICP-OES	5	500 200	100 40	0.047–0.54	This work

^1^ 5-benzyl-4-[4-methoxybenzylideneamino)-4H- 1,2,4-triazole-3-thiol, ^2^ ionic liquid, ^3^ polythionine, ^4^ naphthalene-1-thiol, ^5^ 1-butyl-3-methylimidazolium.

**Table 5:** Analysis of pork liver and kidney and samples spiked with 1.0 mg kg^−1^ Ni, Co and Cr, 5.0 mg kg^−1^ Pb, Cd, Mn and Cu, and 50.0 mg kg^−1^ Zn and Fe; *n* = 6.

Analite	Found, mg kg^−1^	Spiked Pork Liver, mg kg^−1^	Recovery, %	RSD, %	Found, mg kg^−1^	Spiked Pork Kidney, mg kg^−1^	Recovery, %	RSD, %
	Neocuproine/GO							
Cd	1.41 ± 0.01	6.38 ± 0.19	99.4	2.97	2.83 ± 0.09	7.76 ± 0.15	98.6	1.93
Pb	7.31 ± 0.26	12.28 ± 0.33	99.4	2.69	15.65 ± 0.16	20.57 ± 0.38	98.4	1.85
Zn	156 ± 3	205 ± 3	98	1.46	62.03 ± 2.83	110 ± 2	95.9	1.82
Fe	208 ± 3	258 ± 4	99	1.55	138 ± 2	185 ± 3	94	1.62
Mn	6.39 ± 0.06	11.19 ± 0.17	96	1.52	3.15 ± 0.08	7.98 ± 0.12	96.6	1.50
	Batocuproine/GO							
Cd	1.32 ± 0.04	6.29 ± 0.09	99.4	1.43	2.45 ± 0.018	7.49 ± 0.02	101	0.27
Pb	5.97 ± 0.08	10.68 ± 0.23	94.2	2.15	14.81 ± 0.17	19.63 ± 0.13	96.4	0.66
Zn	155 ± 2	204 ± 2	98	0.98	67.47 ± 0.24	118 ± 1	101	0.85
Fe	203 ± 4	251 ± 3	96	1.19	135 ± 3	184 ± 2	98	1.09
Mn	6.80 ± 0.081	11.74 ± 0.17	98.8	1.45	3.68 ± 0.076	8.70 ± 0.11	100.4	1.26
Cr	0.22 ± 0.002	1.19 ± 0.002	97	0.17	0.16 ± 0.001	0.64 ± 0.005	96	0.78
Cu	25.15 ± 0.21	30.06 ± 0.38	98.2	1.26	9.5 ± 0.036	14.28 ± 0.23	95.6	1.61
Co	0.23 ± 0.004	1.24 ± 0.003	101	0.24	0.23 ± 0.002	0.74 ± 0.007	102	0.95
Ni	0.12 ± 0.003	1.09 ± 0.002	97	0.18	0.51 ± 0.005	1.49 ± 0.003	98	0.20

## Data Availability

Not applicable.

## Sample Availability

Samples of the compounds are not available from the authors.
